# Genome-Wide Profiling of Endogenous Single-Stranded DNA Using the SSiNGLe-P1 Method

**DOI:** 10.3390/ijms241512062

**Published:** 2023-07-27

**Authors:** Dongyang Xu, Yu Huang, Lingcong Luo, Lu Tang, Meng Lu, Huifen Cao, Fang Wang, Yong Diao, Liudmila Lyubchenko, Philipp Kapranov

**Affiliations:** 1 Institute of Genomics, School of Medicine, Huaqiao University, 668 Jimei Road, Xiamen 361021, China; xudongyang@hqu.edu.cn (D.X.);; 2 National Medical Research Center for Radiology, Ministry of Health of Russia, 125284 Moscow, Russia; 3 State Key Laboratory of Cellular Stress Biology, School of Life Sciences, Xiamen University, Xiamen 361102, China

**Keywords:** endogenous single-stranded DNA, P1 endonuclease, mitochondrial DNA replication, replication origin, R-loop, promoter

## Abstract

Endogenous single-stranded DNA (essDNA) can form in a mammalian genome as the result of a variety of molecular processes and can both play important roles inside the cell as well as have detrimental consequences to genome integrity, much of which remains to be fully understood. Here, we established the SSiNGLe-P1 approach based on limited digestion by P1 endonuclease for high-throughput genome-wide identification of essDNA regions. We applied this method to profile essDNA in both human mitochondrial and nuclear genomes. In the mitochondrial genome, the profiles of essDNA provide new evidence to support the strand-displacement model of mitochondrial DNA replication. In the nuclear genome, essDNA regions were found to be enriched in certain types of functional genomic elements, particularly, the origins of DNA replication, R-loops, and to a lesser degree, in promoters. Furthermore, interestingly, many of the essDNA regions identified by SSiNGLe-P1 have not been annotated and thus could represent yet unknown functional elements.

## 1. Introduction

While in vivo DNA exists predominantly in the double-stranded form, various types of endogenous single-stranded DNA (essDNA) regions are also known to exist, including those that are formed at the displacement loop (D-loop) in the mitochondrial genome [1] or telomeres [2]. They also occur during biological processes like replication [3,4], transcription [5,6], the DNA damage response [7], and DNA repair [8] and exist as short DNA species like 7S DNA [1] and other extrachromosomal DNA species [9] or appear on other non-B DNA structures [10]. EssDNA regions can arise from the strand separation of double-stranded DNA (dsDNA) that takes place in replication origins or transcription bubbles [3,4,5,6] from gapped regions of genomic DNA (gDNA) formed during DNA metabolic processes like lagging strand replication [11,12] or from short or free DNA species [1,9]. Some essDNA regions can be viewed as transient intermediates of biological processes, as exemplified by essDNA regions present in transcription bubbles or formed during DNA synthesis in DNA repair or DNA replication [3,4,5,6,8]. However, in other cases, stable essDNA regions can have regulatory functions, as exemplified by the essDNA regions formed at promoters where they can recruit specific proteins and result in the regulation of gene expression [13,14,15,16]. A special case of stable essDNA is represented by DNA–RNA hybrids also known as R-loops that are formed during transcription [16,17,18,19]. At least in some cases, R-loops have been shown to have regulatory functions: they were particularly shown to affect transcription activation and termination, chromatin structure, and epigenetic status [16,17,18,19]. Interestingly, the accumulation of R-loops can lead to telomere lengthening and delayed senescence [16]. In some cases, for example, essDNA regions formed in the mitochondrial D-loop, the function of the essDNA is not known. However, it has been hypothesized that the open conformation of the D-loop might be important for the access of proteins involved in mitochondrial DNA (mtDNA) replication or transcription to the binding sites located in the D-loop region and thus can be involved in the modulation of mtDNA replication and transcription processes [1]. In addition to serving as the intermediates in biological processes and having regulatory functions, regions of essDNA also represent a source of genomic instability as illustrated by the increase in various types of DNA damage in association with R-loops [20,21,22,23,24].

As a result of the involvement of essDNA in multiple key biological processes and its relationship with genomic stability, a number of techniques have been developed to detect and characterize essDNA regions. Some of the methods work in a low throughput manner and can only profile specific loci or detect the abundance of essDNA at the global level without revealing the sequence information [25,26,27,28,29,30]. For example, the quantitative amplification of the single-stranded DNA (QAOS) method relies on the annealing of a tagging primer at a low temperature to essDNA loci followed by PCR amplification [25,26]. This method was used to detect the relative abundance of essDNA in a specific locus using real-time PCR with sequence-specific primers or to quantify essDNA at a global level using degenerate primers followed by agarose gel electrophoresis [25,26]. Although theoretically, the method has the potential to detect yet unknown essDNA regions, this has not been reported in the studies that used the method [25,26]. Some methods used immunostaining of essDNA regions in combination with immunofluorescence microscopy to estimate the accumulation of essDNA [27,28,29,30]. For example, some such methods used the bromodeoxyuridine (BrdUrd) base analog which gets incorporated into the chromosomal DNA and is only accessible to the anti-BrdUrd antibody when the DNA is in single-stranded form [27,28,29]. Other methods relied on the anti-ssDNA antibody MAB3299 to immunostain the essDNA [30].

On the other hand, high-throughput essDNA mapping techniques can identify the exact sequence of essDNA at the genome-wide level based on next-generation sequencing (NGS); however, these studies were usually focused on essDNA involved in specific types of biological processes, such as transcription [5,6], replication [3,31], DNA damage and repair [32], or non-B-form DNA structures [10]. For example, kethoxal-assisted single-stranded DNA sequencing (KAS-seq) captured essDNA in humans and mice through the specific reaction between N3-kethoxal and guanines in essDNA which was then followed by NGS analysis [5]. This study mostly focused on studying the dynamics of RNA polymerase II (RNA Pol II) mediated transcription and on the characterization of enhancers based on the presence of essDNA regions [5].

Another method, ssDNA-seq, employed permanganate treatment that could preferentially oxidize pyrimidine residues in the essDNA to generate oxidized bases that cannot base pair with the complementary strand and thus become susceptible to mung bean nuclease cleavage [6]. The cleaved DNA ends are then tailed with terminal transferase (TdT) in the presence of biotinylated nucleotides, followed by sequencing library construction and NGS [6]. This study found that promoter melting is a key regulatory step of gene expression in eukaryotes [6]. Although the majority of essDNA regions detected with ssDNA-seq were consistent with the RNA Pol II profile and associated with active transcription, the ones not co-localized with RNA Pol II could represent other DNA conformations [6,10]. Therefore, in a subsequent study, the same group combined ssDNA-seq with RNA Pol II binding data and computational analysis of sequence motifs to characterize various forms of non-B DNA structures like Z-DNA and quadruplexes [10]. Another group combined non-denaturing bisulfite treatment, which converts cytosines to uracils in essDNA, with NGS to map and characterize gDNA gaps in *E. coli* grown under different conditions [11]. By combining this method with chromatin immunoprecipitation sequencing (ChIP-seq), the same group later developed the ssGap-seq method to detect the binding of RecA and essDNA binding proteins to essDNA in *E. coli* [12].

While powerful, the existing high-throughput techniques have their limitations. For example, the essDNA profiles identified by KAS-seq strongly correlate with those of RNA Pol II—a feature that could be advantageous if one is studying nascent transcription but also represents a potential disadvantage if other essDNA regions are of interest [5]. The ssDNA-seq method requires relatively large amounts of cells (~8 × 10^7^), thus limiting the potential utility of this method [6]. Therefore, here, we present a proof-of-principle development of another essDNA mapping method which combines the “single-strand break mapping at nucleotide genome level” (SSiNGLe) method previously developed by our group to detect DNA single-strand breaks (SSBs) [33] and in-situ digestion of crosslinked nuclei with P1 endonuclease, which preferentially digests single-stranded DNA or RNA while having a much lower activity on dsDNA [34]. Using this method, which we call SSiNGLe-P1, we investigated the patterns of essDNA regions in both the mitochondrial and nuclear genomes.

## 2. Results

### 2.1. Concept of SSiNGLe-P1: A P1 Endonuclease-Based Strategy for the Detection of essDNA Regions

**Figure 1:**
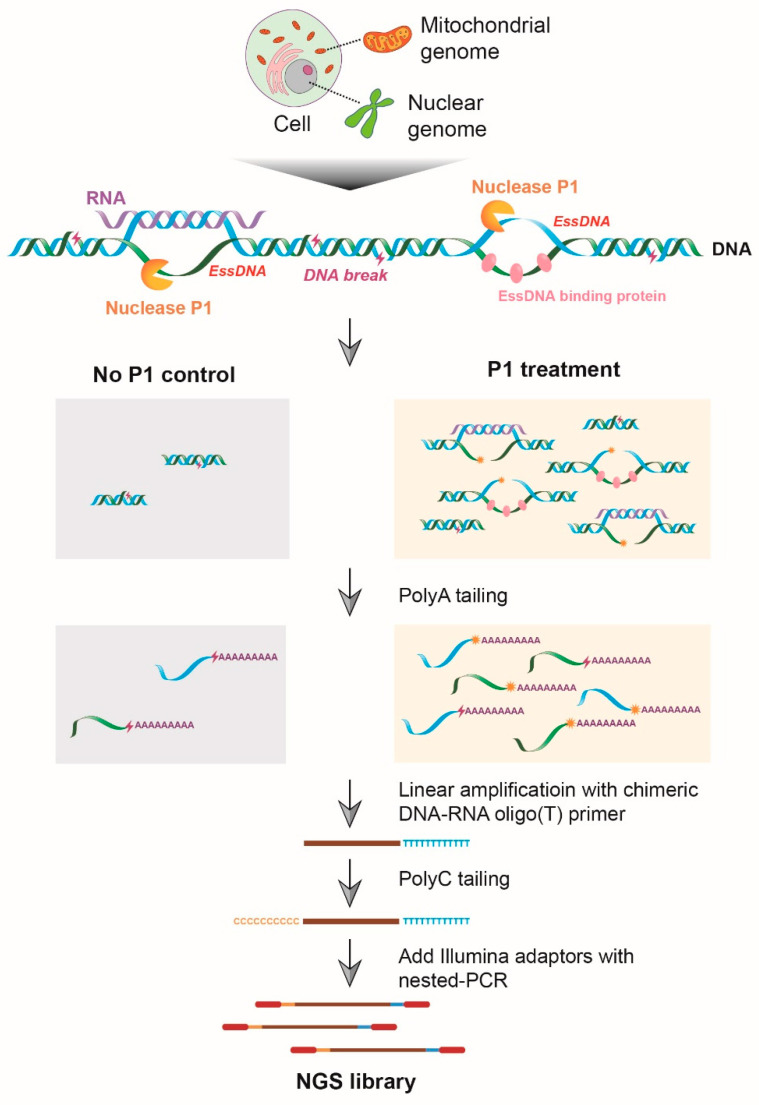
Diagram illustrating the wet lab part of SSiNGLe-P1. Crosslinked nuclei are subjected to limited digestion with P1 endonuclease that generates 3′-OH termini from endogenous single-stranded DNA (essDNA). The samples that are not treated with P1 serve as the control for the endogenous background of DNA breaks with 3′-OH termini. The 3′-OH termini are tailed with polyA by TdT. The tailed DNA molecules are linearly amplified with the chimeric DNA–RNA oligo(T) primer. The products of linear amplification are tailed with polyC using TdT. The appropriate adaptors are then added with nested PCR and the resulting library is subjected to next-generation sequencing (NGS).

The method presented here is based on initial limited P1 digestion of crosslinked nuclei that generates 3′-OH termini in the essDNA regions followed by the precise identification of the genomic sites of the P1-generated termini using SSiNGLe (Figure 1). Briefly, the 3′-OH termini are tailed with polyA by TdT; then, a chimeric DNA–RNA oligo (T) primer is used to linearly amplify the sequence with polyA tags, the products of which are then tailed with polyC. The polyA and polyC tags are used as the anchor sequences for the construction of the NGS library (Figure 1). The genomic coordinates of the 3′-OH termini are then inferred from the genomic coordinates of aligned SSiNGLe-generated NGS reads. We established both the experimental and analytical pipeline for essDNA detection (Figure 1 and Figure S1) and named the method SSiNGLe-P1 (SSiNGLe-P1-based essDNA detection).

Since over-digestion with P1 can break an entire essDNA region down to mononucleotides [35], as the first step in the method development we performed a titration experiment in which we tested various amounts of P1 on the cross-linked nuclei from the human leukemia K562 cell line: 0.01, 0.1, 1, and 10 units (U). Since background 3′-OH termini, derived from SSBs [33,36] or the lagging-strand replication [37], would be also detectable by SSiNGLe-P1, we also used samples without the P1 treatments as controls. As shown in Figure S2, when equal or less than 1 U of P1 is used, the high molecular weight gDNA remained intact but increasing the P1 amount to 10 U led to its visible degradation which is consistent with the low yet measurable activity of P1 towards dsDNA [38]. Nonetheless, the samples treated with the highest (10 U) amount of P1 could represent an additional negative control since it would be expected that essDNA regions would be either totally degraded or depleted under these conditions. Therefore, in all analyses below, we used the samples generated with different amounts of P1 to identify the true essDNA regions.

### 2.2. Application of SSiNGLe-P1 to Evaluate the Strand-Displacement Model of mtDNA Replication

**Figure 2:**
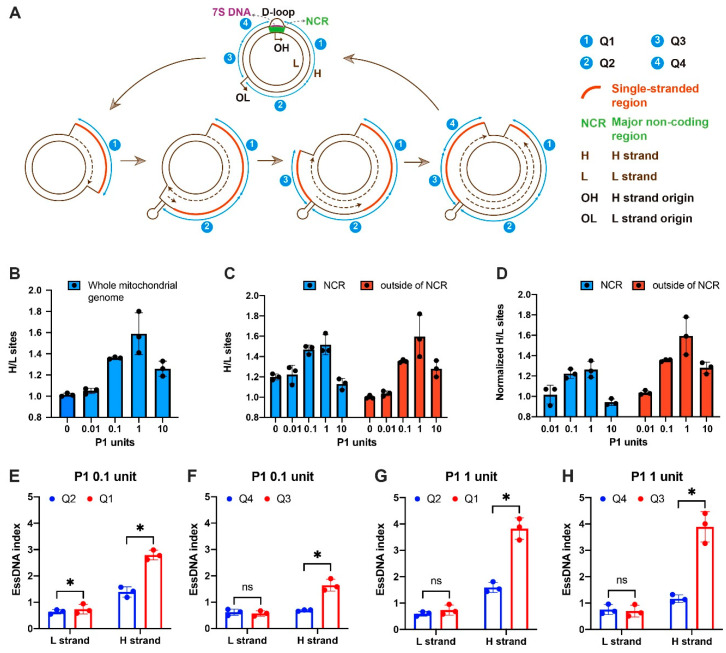
Detection of essDNA in the mitochondrial genome. (**A**) Illustration of the strand-displacement model of mitochondrial DNA replication. The replication is initiated at the H-strand origins located in NCR to produce the leading strand H-strand using the L strand as the template. When the replication progresses to approximately two-thirds of the mitochondrial genome, the L strand origin (OL) is exposed on the parental H strand to form a stem-loop structure and L strand replication starts using the parental H strand as the template. The mitochondrial genome outside of NCR and the stem-loop of OL is split into four regions: Q1–Q4. (**B**) The ratio of 3′-OH sites with unique genomic coordinates on the H vs. the L strands of the entire mitochondrial genome. (**C**,**D**) The ratio and normalized ratio of 3′-OH sites with unique genomic coordinates on the H vs. the L strands inside and outside of NCR. The normalized ratio was obtained by dividing the H/L ratios of the P1 treated samples to the H/L ratio of the untreated control. (**E**–**H**) The essDNA indexes were obtained using 0.1 U (**E**,**F**) or 1 U (**G**,**H**). Asterisks indicate significant differences under two-sided paired Student’s *t*-test (*p* value < 0.05), while “ns” means no statistically significant difference.

The mechanisms of replication of the circular mammalian mtDNA were studied for decades. The strand-displacement model is the most widely accepted model of mtDNA replication; however, the inability of the model to explain experimental evidence found in some studies has led to some controversies in the field and to alternative models of mtDNA replication [39,40,41,42,43]. Based on the strand-displacement model, the nascent heavy (H) strand is made first using the light (L) strand as the template, therefore, the parental H strand should exist in the single-stranded form due to the asynchronous nature of mtDNA replication (Figure 2A) [39]. On the other hand, under this model, the L strand should mostly remain double-stranded (Figure 2A) [39]. Thus, the existence of extensive regions of essDNA biased towards the H-strand is a distinct hallmark feature of the strand-displacement model (Figure 2A) [39]. However, this feature does not exist in the major alternative strand-coupled model [40,41]. Therefore, SSiNGLe-P1 is well-suited to determine if such H-strand-biased regions of essDNA exist and we thus used the method to further explore the validity of the strand-displacement model.

We first mapped the NGS reads to each strand of the mitochondrial genome and then calculated the ratio of P1-derived sites with unique coordinates that mapped to the H vs. the L strand in the K562 samples treated with different amounts of P1 (Figure 2B, Table S1). For this purpose, we used mitochondria that co-purified with crosslinked nuclei and unbroken cells obtained following lysis of the plasma membrane and low-speed centrifugation. Using this approach, we could detect strong mitochondrial signals in our previous SSB genome profiling study [33]. With the increasing amount of P1, the H vs. L strand ratio increased and peaked at 1 U of P1 and then dropped in the 10 U treatment (Figure 2B, Table S1). This profile was consistent with the expectation that the H strand is more sensitive to P1 and thus is more single-stranded than the L strand. Also, as expected, the H/L ratio dropped in the 10 U sample due to the non-specific activity of P1. To confirm the essDNA bias on the H-strand, we then calculated the H/L ratios of the essDNA signal separately inside and outside the boundaries of the non-coding region (NCR), which contains the D-loop, a known essDNA region. This was performed to ensure that our data are not affected by the known essDNA regions formed in the D-loop. Furthermore, we calculate the H/L ratios in three different ways to avoid potential analytical bias: (1) all termini were counted, in which case termini at the same location were counted multiple times, (2) only unique sites representing 3′ termini with unique genomic coordinates were counted, and (3) the average depth of sites was calculated by dividing the total numbers of termini by the total numbers of the corresponding unique sites (Figure S3). In mitochondrial NCR, even in the untreated sample, the ratio of all termini on the H versus L strand was >50, reflecting the background 3′-OH termini signal caused by the 3′ termini of the 7S DNA species (Figure S4A) [1]. However, as expected, the H/L ratio became significantly reduced when sites with unique genomic coordinates were used due to the removal of redundant signal coming from the 7S DNA 3′ termini (Figures 2C and S4A). With the increase in the P1 amount, the H/L ratio increased and peaked at 1 U of P1 and then dropped in the 10 U treatment (Figure 2C), suggesting that the method can detect the essDNA that is known to exist in the D-loop.

To further remove the contribution from background termini, the ratios were also normalized to the untreated control to obtain the normalized ratios. Similarly to the trend observed in the whole H strand (Figure 2B, Table S1), in regions both inside and outside of the NCR, the H strand showed a higher P1-derived signal than the L strand at all three counting approaches (Figures 2C,D and S4A–D, Table S2). Interestingly, the peak of the H vs. L ratio was higher in regions outside of the NCR (Figure 2C,D), which could potentially be explained by the presence of an R-loop in this region (see Discussion). Overall, at the whole mitochondrial genome level, our data support the strand displacement model.

We then explored the single-stranded status of the mitochondrial genome by dividing the regions outside of NCR and the stem-loop structure of OL into four regions: Q1–Q4, which should have different levels of essDNA bias towards the H-strand based on the strand-displacement model (Figure 2A). The replication of the L strand starts using the nascent H strand as the template only when the synthesis of the nascent H strand passes the L strand replication origin OL [39] (Figure 2A). Therefore, the H strand of Q1 should exist in the single-stranded form longer than that of Q2. Using a similar logic, the synthesis of the L strand should finish first in Q4 and then in Q3. Therefore, the H strand of Q3 should exist in the single-stranded form longer than that of Q4.

In this analysis, we profiled the single-stranded status of the H and L strands separately and used the status of the L strand as a control. Therefore, instead of the H vs. L ratio, we calculated the essDNA index for each strand separately, which is defined as the ratio of the normalized read count between the P1-treated samples and the untreated control as an indication of the P1 sensitivity and hence the single-stranded status. Since 0.1 and 1 U can most effectively generate essDNA termini in regions outside of the NCR (Figures 2C,D and S4A–D, Table S2), we used these concentrations to calculate the essDNA index. With both P1 concentrations, the Q1 and Q3 essDNA indices of the H strand were always higher than 1, indicative of their sensitivity to P1 and the existence of essDNA (Figure 2E–H, Table S3). The essDNA indices of Q1 and Q3 in the H strand were significantly higher than the corresponding indices ofQ2 and Q4, respectively, (Figure 2E–H, Table S3). In contrast, no significant differences were observed for the L strand with the exception of the Q1 vs. Q2 comparison in the 0.1 U digestion condition (Figure 2E, Table S3). However, the L strand indices were always ≤1, indicating no P1 sensitivity (Figure 2E, Table S3). Overall, these results are consistent with the strand-displacement model and thus provide new supporting evidence for it. On the other hand, these data also strongly argue that the SSiNGLe-P1 method can detect essDNA and can be used to characterize the dynamics of essDNA that occur during mtDNA replication.

### 2.3. Detection of essDNA Regions in the Nuclear Genome

**Figure 3:**
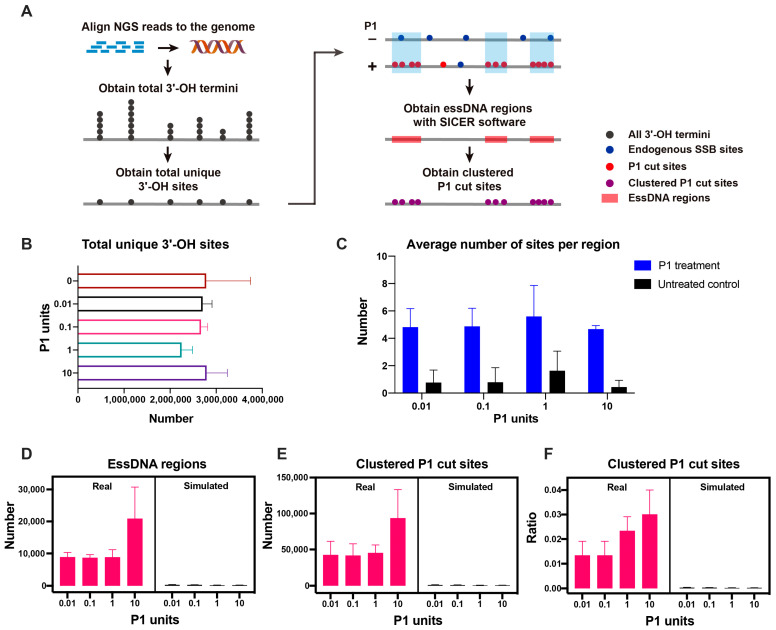
Overview of the essDNA regions detected in the nuclear genome. (**A**) Overview of the analytical pipeline used to obtain the essDNA regions using SICER software. (**B**) Numbers of total 3′-OH termini in each sample type. (**C**) Average numbers of sites found in the essDNA regions in the P1-treated and control samples. (**D**–**F**) The number of essDNA regions (**D**) and clustered P1 cut sites (**E**) and the ratio of clustered P1 cut sites (**F**) in real and simulated samples obtained by the SICER analysis. The error bars indicate the SD of three independent biological replicates.

As the next step, we explored the profile of the essDNA regions in the human nuclear genome (Figure 3A). We detected, on average, 2,254,112–2,795,945 sites with unique genomic coordinates under different P1 digestion conditions (Figure 3B, Table S4). These sites were represented by the termini generated by the P1 activity as well as those that correspond to the background of endogenous SSBs. In contrast to the continuous single-stranded region in the mitochondrial genome represented by the entire H-strand, the P1-derived termini derived from essDNA regions in the nuclear genome would be expected to form discrete clusters. Therefore, to remove the contribution of the background SSBs and identify essDNA regions, we used the SICER analytical tool [44] to obtain the essDNA regions enriched for P1 cut sites by using the P1-treated samples as the input and the untreated samples as the control (Figure 3A). As a result, on average there were 4.7 to 5.6 sites per region in samples treated with 0.01–10 U of P1, which as expected were higher than the 0.4 to 1.6 sites in each of the corresponding untreated controls (Figure 3C, Table S4). On average, 8626–20,837 essDNA regions were obtained for each P1 amount used (Figure 3D and Table S4). The sites of the P1-derived termini located in the essDNA regions were named as clustered P1 cut sites and used for downstream analyses. The average number of such sites ranged from 41,567 to 93,406 (Figure 3E, Table S4). Overall, the clustered P1 cut sites represented a small fraction, ~1.5% to ~3.3%, of the total sites detected in the corresponding samples (Figure 3F, Table S4), which probably reflected both the high stringency of the cluster-based method as well as the relatively high background from the endogenous SSBs (see Discussion).

To evaluate the background noise during the generation of essDNA regions, we performed 100 simulation experiments for each replicate of each of the P1 unit amounts (including the control samples) using randomly-generated data (see Materials and Methods). In this analysis, the numbers of the simulated essDNA regions, clustered P1 cut sites, and the ratio of clustered P1 cut sites were much lower than those found in the real data (Figure 3D–F, Table S5). For example, the fraction of simulated clustered P1 cut sites vs. those obtained in the real experiments was almost always below 4% (Table S5). Overall, the simulation experiments indicated that background noise was low in the essDNA regions detected by SICER.

### 2.4. Properties of the Nuclear essDNA Regions Identified by SSiNGLe-P1

We then explored the distribution of the essDNA regions with respect to various classes of genomic elements that we subdivided into two groups. One group contained genomic elements that were either expected to contain essDNA, such origins of DNA replication [45,46,47,48] and R-loops [18], or where essDNA regions have sometimes been previously found, such as promoters [5]. Another group contained other annotated genomic elements, such as enhancers, insulators, exons, and introns. To estimate the enrichment, we calculate the odds ratios of the enrichment of the clustered P1 cut sites (Materials and Methods).

**Figure 4:**
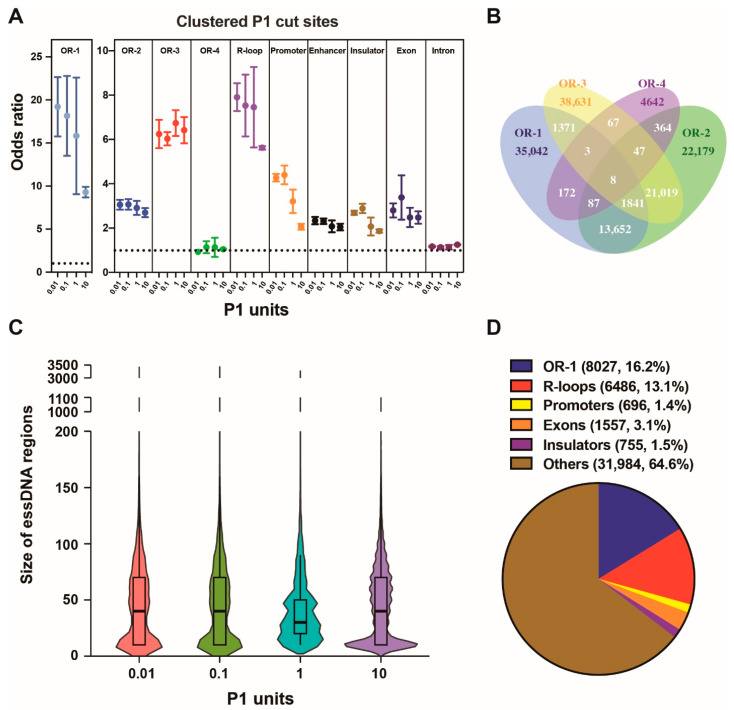
Properties of the essDNA regions in the nuclear genome identified by SSiNGLe-P1. (**A**) Odds ratio of the clustered P1 cut sites in various genomic elements obtained in the samples treated with the indicated amount of P1 (*x*-axis). Error bars indicate the SD of three independent biological replicates. (**B**) Venn diagram showing overlap among the four origin databases. (**C**) Size distribution of the nuclear essDNA regions found in this work. (**D**) Fraction of the essDNA regions overlapping various genomic elements. The overlap was performed using a hierarchical strategy based on the order of the listed genomic elements from top to bottom, i.e., the essDNA regions were first overlapped with the “OR-1” origins and then the ones that did not overlap with the origins were overlapped with the R-loops and so on.

We used four databases of replication origins (named as “OR-1”, “OR-2”, “OR-3”, and “OR-4”, respectively) found by previous studies in K562 [45,46,47,48], the same cell line used in the current work. The “OR-1” database was obtained using a ChIP-seq strategy to map binding sites of the origin recognition complex 2 (ORC2) protein, which is a subunit of the origin recognition complex [45]. The origins in the “OR-2”, “OR-3”, and “OR-4” databases were obtained using a different approach, which was based on various techniques to identify nascent DNA strands [46,47,48]. The odds ratios increased significantly in the clustered P1 cut sites for the origins from all 3 databases (Figure 4A). The highest odds ratios were observed with the origins from the “OR-1” database reaching 19.22 in the 0.01 U sample (Figure 4A, Table S6). Furthermore, only the “OR-1” origins have shown a marked decrease in the enrichment odds ratio in the high 10 U P1 amount (Figure 4A). Somewhat similar profile, albeit with smaller enrichment odds ratios and smaller decrease of the odds ratios in the 10 U samples, was shown by the “OR-2” origins (Figure 4A). Interestingly, we found no enrichment in the “OR-4” database, while the characteristic depletion in the 10 U P1 sample was absent in the “OR-3” database (Figure 4A).

Strikingly, the differences in the SSiNGLe-P1 profiles, observed among the four different datasets apparently representing the same type of genomic element (origins of replication) and obtained from the same cell type, made us investigate the overlap between the four different datasets of origins in more detail. Surprisingly, as shown in Figure 4B, the overlap between the four datasets was not high. For example, the majority of origins in the “OR-1” database, 67.16% (35,042/52,176), were not detected in any of the other three datasets (Figure 4B). The corresponding fractions were 37.47% (22,179/59,197), 61.33% (38,631/62,987), and 86.12% (4642/5390) for the “OR-2”, “OR-3”, and “OR-4” databases (Figure 4B). Therefore, the four different origin datasets were significantly different from each other. Interestingly, consistent with the results from SSiNGLe-P1, the “OR-3” and “OR-4” databases showed very small overlap with “OR-1” of 5.12%, (3223/62,987) and 5.01% (270/5390), respectively, while “OR-2” had a much higher overlap (26.33%, 15,588/59,197), as shown in Figure 4B. Altogether, based on the analysis of the SSiNGLe-P1 profiles in different P1 treatment conditions, the regions in the “OR-1” database most likely represent the true origins of replication while the majority of the ones in the other three databases, especially “OR-3” and “OR-4”, likely correspond to false positives. However, we cannot totally exclude the possibility that the apparently false positive regions in the “OR-2”, “OR-3”, and “OR-4” databases represent true origins, features of which are different from those of the “OR-1” and SSiNGLe-P1-detected origins.

Interestingly, in R-loops, we observed a trend very similar to that found in “OR-1”, also consistent with the known essDNA regions in R-loops (Figure 4A). The maximum enrichment odds ratio in R-loops reached 7.91 in the 0.01 U P1 sample (Figure 4A, Table S6). Furthermore, we observed a similar trend, albeit with lower enrichment ratios in the promoters, where the maximum enrichment odds ratio reaches 4.40 in the 0.1 U P1 sample (Figure 4A, Table S6). We could observe some lower odds ratios in the insulators and exons; however, the general pattern of drop in the 10 U P1 sample could still be observed in these elements. Yet, while we observed some enrichment in the enhancers, we did not observe a marked drop in the 10 U sample, arguing against specific enrichment of the essDNA regions in these elements (Figure 4A). Interestingly, as shown in Figure 4C and Table S7, the sizes of the essDNA regions found in this work were larger than those present in transcriptional bubbles and typically range from 14 to 22 nt [49]. For example, under the 0.01 U and 0.1 U of P1, the essDNA region size ranged from 10 bp to 3410 bp, with the median size of 40–50 bp (Figure 4C, Table S7). These results suggest that the majority of essDNA regions detected here do not represent transcription bubbles, in contrast to some existing studies that focused on essDNA regions generated during transcription [5,6]. While it is not clear if the methods used in these studies preferentially detected essDNA in transcription bubbles, these differences suggest that different methods are needed in order to fully capture different types of essDNA in different biological processes.

Since the odds ratios of overlap between the clustered P1 cut sites and the genomic elements were the highest in the samples treated with 0.01 U and 0.1 U of P1 and then dropped with increasing amounts of P1 (Figure 4A, Table S6), we defined the essDNA regions as the merged region of those obtained with either 0.01 U or 0.1 U of P1. In total, we defined 49,505 essDNA regions by merging the regions found in samples treated with either 0.01 U or 0.1 U of P1 (Table S8). As can be seen in Figure 4D and Table S9, approximately one third of the essDNA regions mapped to the “OR-1” origins (16.2%) or R-loops (13.1%). An additional 1.4% mapped to the annotated promoters (Figure 4D). Minor fractions of the essDNA regions were located in exons (3.1%) and insulators (1.5%) (Figure 4D). Thus, over a half (64.6%) of essDNA regions detected in this study were not associated with the above types of genomic elements (Figure 4D), potentially indicating the existence of other types of essDNA regions or additional origins of replication or R-loops not identified by the previous assays (see Discussion).

### 2.5. The essDNA Regions Tend to Occur in Promoters of Highly Expressed Annotated Genes

**Figure 5:**
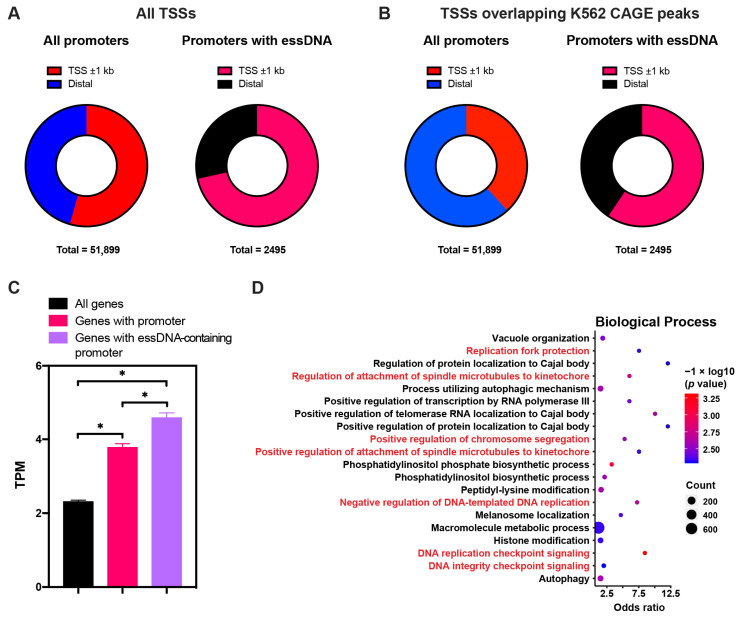
Characterization of essDNA-containing promoters. (**A**,**B**) Distribution of all the essDNA-containing promoters within (proximal) or outside of (distal) ±1 kb regions around the annotated transcriptional start sites (TSSs). (**C**) Expression levels of all protein-coding genes: those with all promoters and those with essDNA-containing promoters. Error bars indicate SD of two independent biological replicates. Asterisks indicate a statistically significant difference with *p* value < 0.05 under the two-sided paired Student’s *t*-test. (**D**) Top 20 enriched Gene Ontology (GO) terms found in the genes with essDNA containing promoters. The GO terms related to DNA replication are marked in red.

Since promoter-associated essDNA regions were previously shown to have functional significance for the regulation of gene expression, we further characterized them. Overall, we found that 2495 promoters previously identified in the K562 cell line based on chromatin segmentation [50,51] had an overlap with essDNA regions found in this work. We first asked whether these promoters were associated with the annotated transcriptional start sites (TSSs). Overall, 54.5% (28,235/51,899) of promoters previously found in K562 were located proximal (within ±1 kb) to the annotated TSSs (Figure 5A, Table S10). The corresponding fraction for the promoters containing essDNA regions was higher (1792/2495, 71.8%), which was significant with *p* < 1 × 10^−5^ in a chi-square test (Figure 5A, Table S10). We next refined this analysis by testing TSSs that overlapped the K562 CAGE peaks [52] and thus represented TSSs that were actually expressed in K562. As shown in Figure 5B and Table S10, less than half (19,831/51,899, 38.2%) of all the promoters were found within ±1 kb of such TSSs, which contrasted with a significantly higher proportion (1482/2495, 59.4%) of essDNA-containing promoters that were close to these TSSs (*p* < 1 × 10^−5^, chi-square test). These results suggest that essDNA containing promoters tend to be more associated with TSSs of annotated and expressed genes than promoters in general.

We further calculated the expression level of genes associated with essDNA-containing promoters. In this analysis, a promoter within ±1 kb of a TSS was assigned to the corresponding gene. As expected, genes associated with promoters found in K562 had significantly higher expression levels in that cell line than all genes (Figure 5C, Table S11). Interestingly, genes associated with essDNA-containing promoters had even higher expression levels than genes with promoters in general, suggesting that the presence of essDNA in promoters positively correlated with gene expression. Furthermore, we found that Gene Ontology (GO) terms related to several biological processes, which strikingly included DNA replication, and were highly enriched among the genes associated with promoters containing essDNA regions (Figure 5D), highlighting that essDNA was preferentially enriched in promoters of specific genes. Overall, these results suggest a more wide-spread involvement of essDNA regions formed at promoters in transcriptional activation.

## 3. Discussion

In this study, we established and validated the high-throughput SSiNGLe-P1 detection method for the detection of essDNA in the human genome. In contrast to most of the relevant studies that are focused on the nuclear genome [5,6,10], this method has been shown to be able to profile the essDNA in both the mitochondrial and nuclear genomes. The former has a very compact and well-characterized circular genome yet the mechanism of replication still has some unresolved issues, as evidenced by the existence of several models of mtDNA replication: the classical strand displacement model as well as other alternative models such as the strand-coupled model [39,40,41]. Profiling of essDNA on the mitochondrial genome with the SSiNGLe-P1 method provided an opportunity to assess the replication models from the essDNA angle. Overall, our observations matched very well with the theoretical predictions from the strand displacement model and thus support a major role of this model in human mtDNA replication. Moreover, the profiling and characterization of essDNA in the mitochondrial genome revealed that SSiNGLe-P1 can be used for the detection of essDNA.

Interestingly, in mitochondrial NCR the maximum normalized signal of P1-treated samples vs. the control was also lower than in the rest of the mtDNA, as measured by all three metrics (all termini, unique sites, or the depth of sites, Figures 2D and S4B,D). This observation could potentially be explained by the previously reported existence of the R-loop in the NCR, where an RNA strand hybridizes with the essDNA H strand in the D-loop region [53]. The RNA/DNA hybrid could protect the D-loop from digestion with P1, leading to a lower essDNA signal detected in that region. Compared to the D-loop, the R-loop in NCR is much less well studied and the biological meaning of it is also unclear [53]. Our results provide supporting evidence for the existence of the mitochondrial R-loop; however, other mechanisms that lead to the coating and protection of the D-loop (for example, by binding to proteins) are also possible.

Profiling of essDNA regions in the nuclear genome revealed very strong enrichment in the origins of DNA replication and R-loops. Both types of genomic elements are expected to have essDNA regions, so in a sense, the enrichment could be viewed as an additional validation of the method. Strikingly, however, we observed strong enrichment of essDNA in only one out of the four published databases of replication origins. Furthermore, even though all four databases were derived from the same cell line, they were also quite different from each other. The most likely reason for this was the differences in the techniques used to generate the four databases: while the “OR-1” database with the most enriched essDNA signal was obtained using ChIP-seq mapping of ORC, the other three databases were obtained by mapping of nascent DNA strands. These results emphasize that different methods aimed at discovery of the same genomic features have widely different performance metrics and that identification of true genomic elements likely requires the combination of different methods. In this case, the combination of SSiNGLe-P1 and ORC ChIP-seq (or other methods) could be used to correctly map origins of DNA replication. Likewise, a combination of SSiNGLe-P1 and R-loop mapping methods could potentially significantly improve the accuracy of detection of these genomic elements.

Furthermore, in this study, we also found enrichment of essDNA in other genomic elements, most notably promoters, which is consistent with previous studies. Promoter-associated essDNA was implicated in the regulation of gene expression [13,14,15,16]. For example, essDNA regions in the DRA promoter can be induced, stabilized, and preferentially bound by the YB-1 protein, leading to transcriptional repression [13]. On the other hand, Puralpha protein can bind G-rich essDNA in the promoter of the PDGF-A gene and activate its expression [14]. Interestingly, the study by Wu et al. has found strong enrichment of essDNA in genome-wide promoters using KAS-seq [5]. In that study, most of the essDNA signals in promoters overlapped with those obtained by RNA Pol II ChIP-seq and were thought to reflect transcription initiation and the pausing of RNA Pol II near the TSSs [5]. Moreover, similarly to our results, the strength of the essDNA signal in promoters and the rest of the gene bodies obtained by KAS-seq dropped with the decrease in gene expression [5]. Furthermore, the study by Kouzine et al. found a strong promoter-associated essDNA signal using ssDNA-seq and proposed that promoter melting is a novel regulatory step of gene expression [6]. Subsequently, the same group found that promoters of certain genes were enriched in essDNA in non-B DNA structures and that these essDNA regions might affect the expression patterns of the genes [10]. Overall, our study suggests that thousands of promoters contain essDNA regions and such promoters tend to be associated with annotated TSSs of known genes and with higher expression of the corresponding genes, indicating potential transcription-activating roles of the essDNA regions genome-wide. Strikingly, based on the GO analysis, our results also provide a potential connection between the presence of essDNA at promoters and DNA replication.

Interestingly, the studies by Wu et al. [5] and Kouzine et al. [6,10] did not mention the enrichment of essDNA regions in origins of DNA replication and R-loops regions, the two genomic elements where our study found the highest enrichment of the essDNA signal. At present, it is not clear if this means that KAS-seq and ssDNA-seq cannot detect essDNA regions in those elements or if they have not been included in the analysis in these studies [5,6,10]. Also, while the studies by Wu et al. [5] and Kouzine et al. [6] described the occurrence of essDNA at enhancers, we did not observe obvious enrichment of essDNA at these genomic elements. Although we did observe some enrichment of clustered P1 cut sites in those elements, this enrichment was lower than what was found in the promoters and it did not decrease in response to the high amounts of P1, which result in non-specific cleavage. The latter fact also emphasizes the limitation of SSiNGLe-P1: since this method depends on the enzymatic treatment, the amount of the enzyme used under specific reaction conditions needs to be established and different P1 amounts would likely have to be used in the experiment. Furthermore, different essDNA regions may have different accessibility to P1 depending on the chromatin environment and interaction with other molecules and this feature would represent another potential limitation of our method.

The majority of the essDNA regions found with SSiNGLe-P1 could not be explained by the published essDNA-associated regions. It is almost certain that at least some of those represent origins of DNA replication and/or R-loops that were missed in previous surveys. In addition, they could represent other types of essDNA, such as extrachromosomal essDNA species [9], or diverse forms of non-B DNA structures, such as Z-DNA, G-quadruplex, H-DNA, cruciform, and Stress-Induced Duplex Destabilized DNA (SIDD) [10]. Furthermore, they could also represent totally novel types of essDNA and functional genomic elements. In this work, we identified essDNA regions that are most likely common to multiple cells and are thus more likely to present functionally relevant DNA elements. However, the bulk analysis used in this study would likely hide cell-to-cell variation or cell-specific essDNA regions, the identification of which would require the adaptation of SSiNGLe-P1 to single-cell level analysis. Overall, the functions and biological relevance of the essDNA regions newly discovered using SSiNGLe-P1 require further investigation.

## 4. Materials and Methods

### 4.1. Cell Culture

Human chronic myeloid leukemia cell line K562 (Cell Bank of Chinese Academy of Sciences, Shanghai, China) was maintained in RPMI 1640 (Gibco, Billings, MT, USA) supplemented with 10% heat-inactivated fetal bovine serum (ExCell Bio, Montevideo, Uruguay) and 1% penicillin-streptomycin (Gibco) and cultured at 37 °C in 5% CO_2_.

### 4.2. SSiNGLe-P1: Wet Lab

First, one million K562 cells were crosslinked with 1% formaldehyde (VWR Life Science AMRESCO, Radnor, PA, USA) and lysed to isolate the nuclei pellet with a standard low-speed centrifugation (1500× *g*), as described previously [33], that was later used as the source of DNA for both nuclear and mitochondrial genome analyses. Second, the digestion of essDNA regions in the genome was performed as follows: the pellet was incubated in 320 µL 0.2% SDS buffer (Life Technologies, Carlsbad, CA, USA) for 10 min at 62 °C and quenched with 50 µL of 10% Triton X-100 (Sinopharm Chemical Reagent Co., Ltd, Shanghai, China). The nuclei were collected by centrifugation at 4000 rpm at 27 °C for 10 min and washed twice with 500 µL 1× NEBuffer 1.1 (NEB, Ipswich, MA, USA). After aspiration of the supernatant, the nuclei were resuspended in 50 µL 1× NEBuffer 1.1 (NEB) containing different amounts of the Nuclease P1 (NEB) (0.01 U, 0.1 U, 1 U, and 10 U) at 37 °C for 10 min, followed by inactivation at 75 °C for 10 min. Third, DNA was isolated from the P1 digested samples as described previously [33] and purified with 2× volumes of VAHTS DNA Clean Beads (Vazyme, Nanjing, China) to a final volume of 20 µL. The purified DNA was examined with 1% agarose gels. Fourth, NGS sequencing libraries were constructed with the SSiNGLe-ILM method developed by us previously [33], using 100 ng of DNA as the input. NGS was performed on the Illumina sequencing platform HiSeq X Ten using the paired-end 150 bp (PE150) strategy and outsourced to Novogene Corporation (Beijing, China). Three biological replicates were performed for samples treated with each P1 amount as well as the control samples on a 5-gigabase (GB) scale.

### 4.3. SSiNGLe-P1: Bioinformatics

The analytical pipeline for the detection of essDNA is summarized in Figure S1. Raw reads were filtered to obtain clean reads by removing read pairs with the following features: (1) paired-end reads containing adaptor sequence, (2) the fraction of N bases in either read exceeds 10%, and (3) the fraction of low-quality bases (Q ≤ 5) exceeds 50%. Only paired-end clean reads, where read 1 started with 9–11 Gs and read 2 started with 11–13 Ts and each base of each read had a Phred quality score > 20 were selected. PolyG and polyT sequences at the beginning of reads 1 and 2, respectively, were then trimmed.

For the analysis of essDNA in the mitochondrial genome, trimmed reads were aligned to the human mitochondrial genome (GRCh37/hg19) [54] using BWA-MEM (v0.7.12) with the default settings. The 3′-OH termini were defined as the first bases after the stretches of the 11–13 Ts in the reads 2 of paired uniquely mapped reads. The read count of each nucleotide was obtained and then normalized to the total number of reads aligned to the mitochondrial genome in that sample. To calculate the essDNA index, regions between the boundaries of NCR and the stem-loop structure of OL were used and divided into 4 regions with the following coordinates in the mitochondrial genome: (1) Q1 (10,593–16,024); (2) Q2 (5160–10,592); (3) Q3 (2869–5158); and (4) Q4 (579–2868).

To detect essDNA regions in the nuclear genome, trimmed reads were aligned to the human genome (including nuclear and mitochondrial genome) and the 3′-OH termini were extracted as described above. To avoid alignments likely arising from internal priming to A-rich sequences in the genome, 3′-OH termini where a fraction of Ts in the 20-base 5′ upstream sequence in the read 2 alignment was >40% were removed from the downstream analysis. Then SICER (v1.1) software [44] was used to obtain essDNA regions using the coordinates of the 3′-OH termini in the P1-treated samples versus those untreated samples with the following parameters: redundancy threshold = 1, window size = 10 bp, fragment size = 1 bp, effective genome fraction = 0.8, gap size = 30 bp, and E-value = 0.01. The SICER analyses were performed on each pair of the P1 treated and untreated biological replicates. In the downstream analysis, merged essDNA regions obtained from the 0.01 U and 0.1 U P1 treated samples were used. The genomic coordinates of essDNA regions can be found in Table S8.

### 4.4. Simulation Analysis

First, for each of the P1-treated and control replicates, we generated the same number of 3′-OH termini as in the corresponding real sample (after the filtration for the endogenous polyA sequences) with random coordinates across the nuclear genome using the “shuffle” function of the BEDTools suite (v2) with the default settings [55]. Second, simulated essDNA regions were generated with SICER software with the same parameters as mentioned above and the positions within the simulated regions were defined as the simulated clustered P1 cut sites. A total of 100 simulation experiments were performed.

### 4.5. RNA-Seq Analysis

The RNA-seq data were previously generated by our group from two biological replicates of the normally grown K562 cells [56]. To calculate the TPM of genes, the raw reads were trimmed with the fastq_quality_trimmer of the FASTX-Toolkit (v0.0.13) software [57] to obtain paired-end reads with a Phred quality score ≥ 20 for each base. The TPM of the genes was calculated based on the annotation file “gencode.v39lift37.annotation.gtf” of the GRCh37/hg19 assembly from release 39 of GENCODE [58] by the RSEM (v1.2.28) software with parameters ‘--bowtie2 --paired-end --strand-specific --no-bam-output’.

### 4.6. General Bioinformatics Analysis

The annotations, unless indicated otherwise, were downloaded from the GRCh37/hg19 assembly of the UCSC Genome Browser [54]. The annotations used in this section are summarized in Table S12. The overlaps between clustered P1 cut sites and the different genomic elements were calculated using the “intersect” function of the BEDTools suite (v2) [55]. The known genes were represented by the “UCSC Genes” database [59]. The databases of replication origins (“OR-1” [45], “OR-2” [46], “OR-3” [47], and “OR-4” [48]) and R-loop [18] were obtained from the corresponding publications. Annotations of promoters, enhancers, and insulators for K562 cell line were obtained from the “Chromatin State Segmentation by HMM from ENCODE/Broad” track [50,51]. Odds ratios for each genomic element in a given sample were calculated with the following formula: odds ratio = EP/(TP × (EL/GL)), where EP is the number of clustered P1 cut sites in a given element class; TP is the total number of clustered P1 cut sites in the sample; EL is the total length of the element class in the genome; and GL is the total length of the genome.

The annotation of all TSSs was obtained from the “GENCODE V39lift37” track, “Comprehensive (wgEncodeGencodeCompV39lift37)” table of the UCSC Genome Browser [58]. To obtain the TSSs overlapping the K562 CAGE peaks, CAGE data of K562 were obtained from the published data of the FANTOM5 consortium (FANTOM Source Names 10454-106G4, 10824-111C5, 10824-111C6, and 10824-111C7) (https://fantom.gsc.riken.jp/5/datafiles/latest/basic/human.cell_line.hCAGE/ (accessed on 24 November 2021)) [48]. The CAGE tags from the 4 files were combined for downstream analysis. For calculation of a statistically significant difference, the chi-square test was used based on the chi-square calculator webserver (https://www.socscistatistics.com/tests/chisquare/default2.aspx (accessed on 3 May 2023)).

The GO analysis for protein-coding genes with essDNA containing promoters was performed using the GOstats package (v2.52.0) [60] in the R environment (package org.Hs.eg.db), genes with essDNA-containing promoters within ±1 kb around the TSSs were used as the input, and genes containing any promoters within the same distance around their TSSs were used as the background.

## 5. Conclusions

In summary, the SSiNGLe-P1 method established in this work represents an additional tool to study essDNA, an important and yet not fully understood component of cellular DNA, in both mitochondrial and nuclear genomes in a high-throughput manner. Our results suggest that in addition to the several known types of genomic elements that contain essDNA, there could be others that have novel functions in human cells. Results from this study also suggest that various detection methods have a preference for the detection of different types of essDNA (and other genomic features like origins of DNA replication), which calls for a combination and cross-validation of different methods in order to fully understand the complexity of essDNA. Additional work is still required to establish a full map of essDNA regions in the human genome that would capture all types of essDNA as well as their dynamics in different biological contexts. However, such a resource would certainly provide a valuable contribution to the annotation of the human genome.

## Data Availability

The raw NGS data were submitted to GEO with the accession number GSE235937. The processed data are presented in Supplementary Tables S1–S12.

## Supplementary Materials

The following supporting information can be downloaded at: https://www.mdpi.com/article/10.3390/ijms241512062/s1.

## References

[1] Nicholls T.J., Minczuk M. (2014). In D-Loop: 40 Years of Mitochondrial 7S DNA. Exp. Gerontol..

[2] Greider C.W. (1999). Telomeres Do D-Loop-T-Loop. Cell.

[3] Feng W., Collingwood D., Boeck M.E., Fox L.A., Alvino G.M., Fangman W.L., Raghuraman M.K., Brewer B.J. (2006). Genomic Mapping of Single-Stranded DNA in Hydroxyurea-Challenged Yeasts Identifies Origins of Replication. Nat. Cell Biol..

[4] Holt I.J., Reyes A. (2012). Human Mitochondrial DNA Replication. Cold Spring Harb. Perspect. Biol..

[5] Wu T., Lyu R., You Q., He C. (2020). Kethoxal-Assisted Single-Stranded DNA Sequencing Captures Global Transcription Dynamics and Enhancer Activity in Situ. Nat. Methods.

[6] Kouzine F., Wojtowicz D., Yamane A., Resch W., Kieffer-Kwon K.-R., Bandle R., Nelson S., Nakahashi H., Awasthi P., Feigenbaum L. (2013). Global Regulation of Promoter Melting in Naive Lymphocytes. Cell.

[7] Bantele S.C.S., Lisby M., Pfander B. (2019). Quantitative Sensing and Signalling of Single-Stranded DNA during the DNA Damage Response. Nat. Commun..

[8] Kowalczykowski S.C. (2015). An Overview of the Molecular Mechanisms of Recombinational DNA Repair. Cold Spring Harb. Perspect. Biol..

[9] Hisano O., Ito T., Miura F. (2021). Short Single-Stranded DNAs with Putative Non-Canonical Structures Comprise a New Class of Plasma Cell-Free DNA. BMC Biol..

[10] Kouzine F., Wojtowicz D., Baranello L., Yamane A., Nelson S., Resch W., Kieffer-Kwon K.-R., Benham C.J., Casellas R., Przytycka T.M. (2017). Permanganate/S1 Nuclease Footprinting Reveals Non-B DNA Structures with Regulatory Potential across a Mammalian Genome. Cell Syst..

[11] Pham P., Shao Y., Cox M.M., Goodman M.F. (2022). Genomic Landscape of Single-Stranded DNA Gapped Intermediates in Escherichia Coli. Nucleic Acids Res..

[12] Pham P., Wood E.A., Cox M.M., Goodman M.F. (2023). RecA and SSB Genome-Wide Distribution in SsDNA Gaps and Ends in Escherichia Coli. Nucleic Acids Res..

[13] MacDonald G.H., Itoh-Lindstrom Y., Ting J.P. (1995). The Transcriptional Regulatory Protein, YB-1, Promotes Single-Stranded Regions in the DRA Promoter. J. Biol. Chem..

[14] Zhang Q., Pedigo N., Shenoy S., Khalili K., Kaetzel D.M. (2005). Puralpha Activates PDGF-A Gene Transcription via Interactions with a G-Rich, Single-Stranded Region of the Promoter. Gene.

[15] Miao Y., Jiang J., Ren Y., Zhao Z. (2013). The Single-Stranded DNA-Binding Protein WHIRLY1 Represses WRKY53 Expression and Delays Leaf Senescence in a Developmental Stage-Dependent Manner in Arabidopsis. Plant Physiol..

[16] Santos-Pereira J.M., Aguilera A. (2015). R Loops: New Modulators of Genome Dynamics and Function. Nat. Rev. Genet..

[17] Ginno P.A., Lott P.L., Christensen H.C., Korf I., Chédin F. (2012). R-Loop Formation Is a Distinctive Characteristic of Unmethylated Human CpG Island Promoters. Mol. Cell.

[18] Sanz L.A., Hartono S.R., Lim Y.W., Steyaert S., Rajpurkar A., Ginno P.A., Xu X., Chédin F. (2016). Prevalent, Dynamic, and Conserved R-Loop Structures Associate with Specific Epigenomic Signatures in Mammals. Mol. Cell.

[19] Allison D.F., Wang G.G. (2019). R-Loops: Formation, Function, and Relevance to Cell Stress. Cell Stress.

[20] García-Rubio M.L., Pérez-Calero C., Barroso S.I., Tumini E., Herrera-Moyano E., Rosado I.V., Aguilera A. (2015). The Fanconi Anemia Pathway Protects Genome Integrity from R-Loops. PLoS Genet..

[21] Schwab R.A., Nieminuszczy J., Shah F., Langton J., Lopez Martinez D., Liang C.-C., Cohn M.A., Gibbons R.J., Deans A.J., Niedzwiedz W. (2015). The Fanconi Anemia Pathway Maintains Genome Stability by Coordinating Replication and Transcription. Mol. Cell.

[22] Hatchi E., Skourti-Stathaki K., Ventz S., Pinello L., Yen A., Kamieniarz-Gdula K., Dimitrov S., Pathania S., McKinney K.M., Eaton M.L. (2015). BRCA1 Recruitment to Transcriptional Pause Sites Is Required for R-Loop-Driven DNA Damage Repair. Mol. Cell.

[23] Zhang X., Chiang H.-C., Wang Y., Zhang C., Smith S., Zhao X., Nair S.J., Michalek J., Jatoi I., Lautner M. (2017). Attenuation of RNA Polymerase II Pausing Mitigates BRCA1-Associated R-Loop Accumulation and Tumorigenesis. Nat. Commun..

[24] Cristini A., Groh M., Kristiansen M.S., Gromak N. (2018). RNA/DNA Hybrid Interactome Identifies DXH9 as a Molecular Player in Transcriptional Termination and R-Loop-Associated DNA Damage. Cell Rep..

[25] Booth C., Griffith E., Brady G., Lydall D. (2001). Quantitative Amplification of Single-Stranded DNA (QAOS) Demonstrates That Cdc13-1 Mutants Generate SsDNA in a Telomere to Centromere Direction. Nucleic Acids Res..

[26] Holstein E.-M., Lydall D. (2012). Quantitative Amplification of Single-Stranded DNA. Methods Mol. Biol..

[27] Kilgas S., Singh A.N., Paillas S., Then C.-K., Torrecilla I., Nicholson J., Browning L., Vendrell I., Konietzny R., Kessler B.M. (2021). P97/VCP Inhibition Causes Excessive MRE11-Dependent DNA End Resection Promoting Cell Killing after Ionizing Radiation. Cell Rep..

[28] Kilgas S., Kiltie A.E., Ramadan K. (2021). Immunofluorescence Microscopy-Based Detection of SsDNA Foci by BrdU in Mammalian Cells. STAR Protoc..

[29] Raderschall E., Golub E.I., Haaf T. (1999). Nuclear Foci of Mammalian Recombination Proteins Are Located at Single-Stranded DNA Regions Formed after DNA Damage. Proc. Natl. Acad. Sci. USA.

[30] Chatzidoukaki O., Stratigi K., Goulielmaki E., Niotis G., Akalestou-Clocher A., Gkirtzimanaki K., Zafeiropoulos A., Altmüller J., Topalis P., Garinis G.A. (2021). R-Loops Trigger the Release of Cytoplasmic SsDNAs Leading to Chronic Inflammation upon DNA Damage. Sci. Adv..

[31] Masuda T., Kono N., Tomita M., Arakawa K. (2019). Strand-Specific Single-Stranded DNA Sequencing (4S-Seq) of E. Coli Genomes. Bio. Protoc..

[32] Buhler C., Borde V., Lichten M. (2007). Mapping Meiotic Single-Strand DNA Reveals a New Landscape of DNA Double-Strand Breaks in Saccharomyces Cerevisiae. PLoS Biol..

[33] Cao H., Salazar-García L., Gao F., Wahlestedt T., Wu C.-L., Han X., Cai Y., Xu D., Wang F., Tang L. (2019). Novel Approach Reveals Genomic Landscapes of Single-Strand DNA Breaks with Nucleotide Resolution in Human Cells. Nat. Commun..

[34] Fujimoto M., Kuninaka A., Yoshino H. (1974). Purification of a Nuclease from *Penicillium Citrinum*. Agric. Biol. Chem..

[35] Volbeda A., Lahm A., Sakiyama F., Suck D. (1991). Crystal Structure of Penicillium Citrinum P1 Nuclease at 2.8 A Resolution. EMBO J..

[36] Caldecott K.W. (2008). Single-Strand Break Repair and Genetic Disease. Nat. Rev. Genet..

[37] Okazaki T. (2017). Days Weaving the Lagging Strand Synthesis of DNA—A Personal Recollection of the Discovery of Okazaki Fragments and Studies on Discontinuous Replication Mechanism. Proc. Jpn. Acad. Ser. B Phys. Biol. Sci..

[38] Fujimoto M., Kuninaka A., Yoshino H. (1974). Substrate Specificity of Nuclease P1. Agric. Biol. Chem..

[39] Falkenberg M. (2018). Mitochondrial DNA Replication in Mammalian Cells: Overview of the Pathway. Essays Biochem..

[40] Holt I.J., Lorimer H.E., Jacobs H.T. (2000). Coupled Leading- and Lagging-Strand Synthesis of Mammalian Mitochondrial DNA. Cell.

[41] Bowmaker M., Yang M.Y., Yasukawa T., Reyes A., Jacobs H.T., Huberman J.A., Holt I.J. (2003). Mammalian Mitochondrial DNA Replicates Bidirectionally from an Initiation Zone. J. Biol. Chem..

[42] Yasukawa T., Reyes A., Cluett T.J., Yang M.-Y., Bowmaker M., Jacobs H.T., Holt I.J. (2006). Replication of Vertebrate Mitochondrial DNA Entails Transient Ribonucleotide Incorporation throughout the Lagging Strand. EMBO J..

[43] Reyes A., Kazak L., Wood S.R., Yasukawa T., Jacobs H.T., Holt I.J. (2013). Mitochondrial DNA Replication Proceeds via a “bootlace” Mechanism Involving the Incorporation of Processed Transcripts. Nucleic Acids Res..

[44] Zang C., Schones D.E., Zeng C., Cui K., Zhao K., Peng W. (2009). A Clustering Approach for Identification of Enriched Domains from Histone Modification ChIP-Seq Data. Bioinformatics.

[45] Miotto B., Ji Z., Struhl K. (2016). Selectivity of ORC Binding Sites and the Relation to Replication Timing, Fragile Sites, and Deletions in Cancers. Proc. Natl. Acad. Sci. USA.

[46] Picard F., Cadoret J.-C., Audit B., Arneodo A., Alberti A., Battail C., Duret L., Prioleau M.-N. (2014). The Spatiotemporal Program of DNA Replication Is Associated with Specific Combinations of Chromatin Marks in Human Cells. PLoS Genet..

[47] Martin M.M., Ryan M., Kim R., Zakas A.L., Fu H., Lin C.M., Reinhold W.C., Davis S.R., Bilke S., Liu H. (2011). Genome-Wide Depletion of Replication Initiation Events in Highly Transcribed Regions. Genome Res..

[48] Hansen R.S., Thomas S., Sandstrom R., Canfield T.K., Thurman R.E., Weaver M., Dorschner M.O., Gartler S.M., Stamatoyannopoulos J.A. (2010). Sequencing Newly Replicated DNA Reveals Widespread Plasticity in Human Replication Timing. Proc. Natl. Acad. Sci. USA.

[49] Fiedler U., Timmers H.T. (2001). Analysis of the Open Region of RNA Polymerase II Transcription Complexes in the Early Phase of Elongation. Nucleic Acids Res..

[50] Ernst J., Kellis M. (2010). Discovery and Characterization of Chromatin States for Systematic Annotation of the Human Genome. Nat. Biotechnol..

[51] Ernst J., Kheradpour P., Mikkelsen T.S., Shoresh N., Ward L.D., Epstein C.B., Zhang X., Wang L., Issner R., Coyne M. (2011). Mapping and Analysis of Chromatin State Dynamics in Nine Human Cell Types. Nature.

[52] The FANTOM Consortium and the RIKEN PMI and CLST (DGT) (2014). A Promoter-Level Mammalian Expression Atlas. Nature.

[53] Holt I.J. (2019). The Mitochondrial R-Loop. Nucleic Acids Res..

[54] Kent W.J., Sugnet C.W., Furey T.S., Roskin K.M., Pringle T.H., Zahler A.M., Haussler D. (2002). The Human Genome Browser at UCSC. Genome Res..

[55] Quinlan A.R., Hall I.M. (2010). BEDTools: A Flexible Suite of Utilities for Comparing Genomic Features. Bioinformatics.

[56] Cao H., Zhang Y., Cai Y., Tang L., Gao F., Xu D., Kapranov P. (2022). Hotspots of Single-Strand DNA “Breakome” Are Enriched at Transcriptional Start Sites of Genes. Front. Mol. Biosci..

[57] Gordon A. FASTX-Toolkit. http://hannonlab.cshl.edu/fastx_toolkit/download.html.

[58] Frankish A., Diekhans M., Jungreis I., Lagarde J., Loveland J.E., Mudge J.M., Sisu C., Wright J.C., Armstrong J., Barnes I. (2021). GENCODE 2021. Nucleic Acids Res..

[59] Hsu F., Kent W.J., Clawson H., Kuhn R.M., Diekhans M., Haussler D. (2006). The UCSC Known Genes. Bioinformatics.

[60] Falcon S., Gentleman R. (2007). Using GOstats to Test Gene Lists for GO Term Association. Bioinformatics.

