# Supplementary material for: Genome-Wide Profiling of Endogenous Single-Stranded DNA Using the SSiNGLe-P1 Method
Source: Int J Mol Sci. 2023 Jul 27;24(15):12062. doi: 10.3390/ijms241512062 (PMC10418711; doi:10.3390/ijms241512062)
Supplement: Supplementary file 1 [file ijms-24-12062-s001.zip › Xu_etal_Supplementary_Information/Xu_etal_Supplementary_figures.pdf]

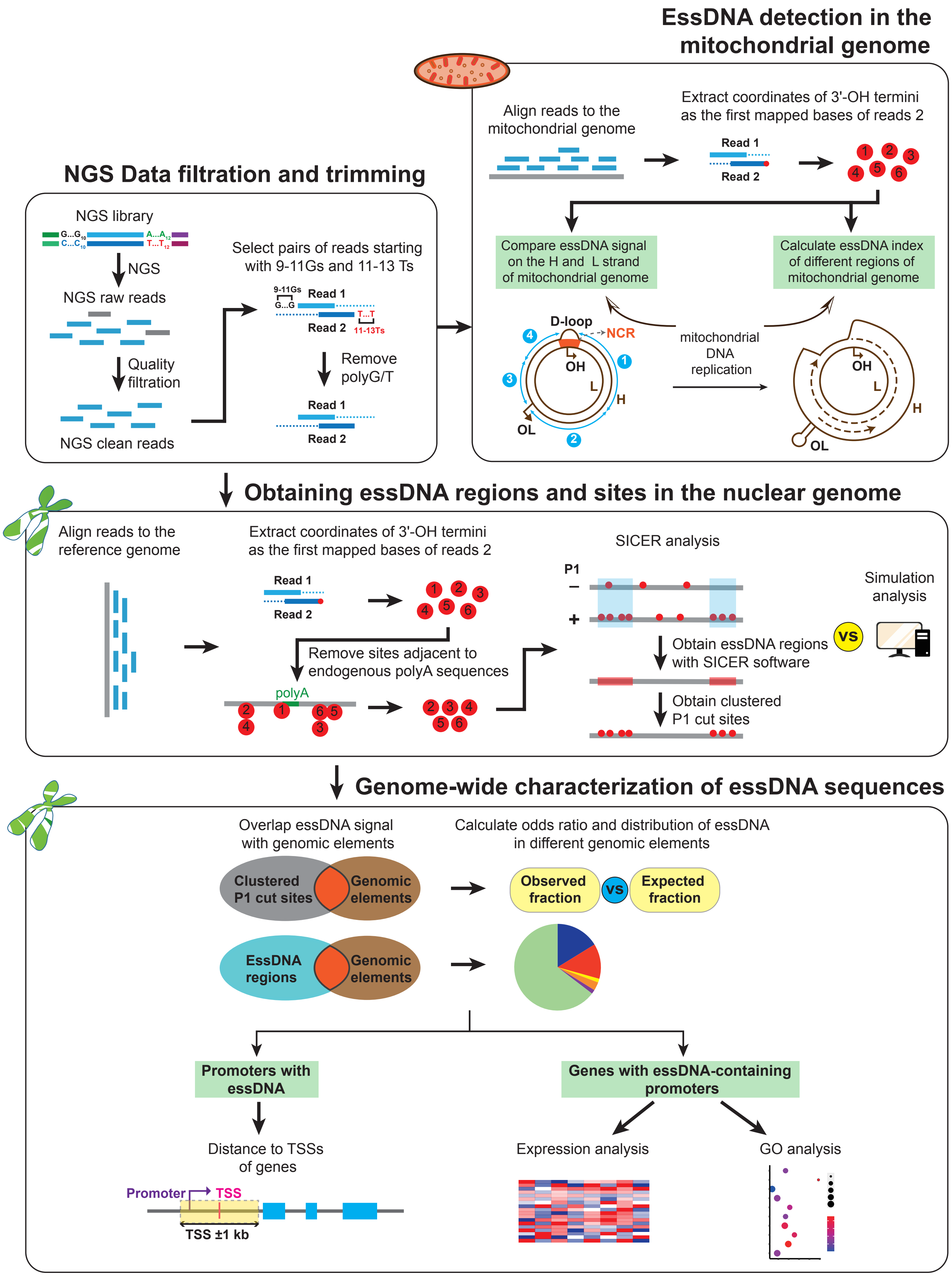

Figure S1. Analytical pipeline used in this work for detection and analysis of the essDNA regions.

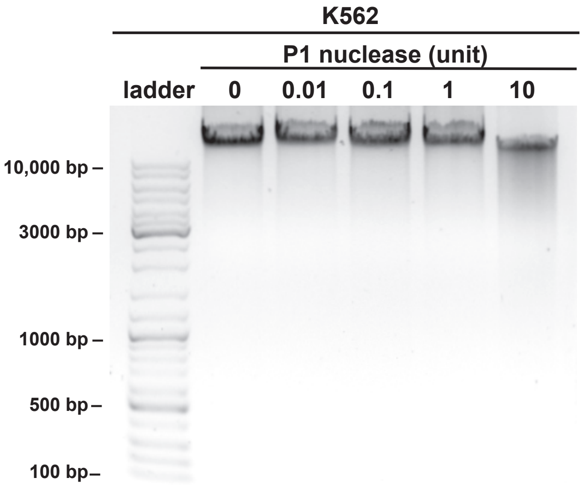

Figure S2. Agarose gel analysis of P1 digested DNA. Genomic DNA isolated from K562 crosslinked nuclei digested with the indicated amounts of P1 is shown. For each treatment, three biological replicates were combined into a single lane.

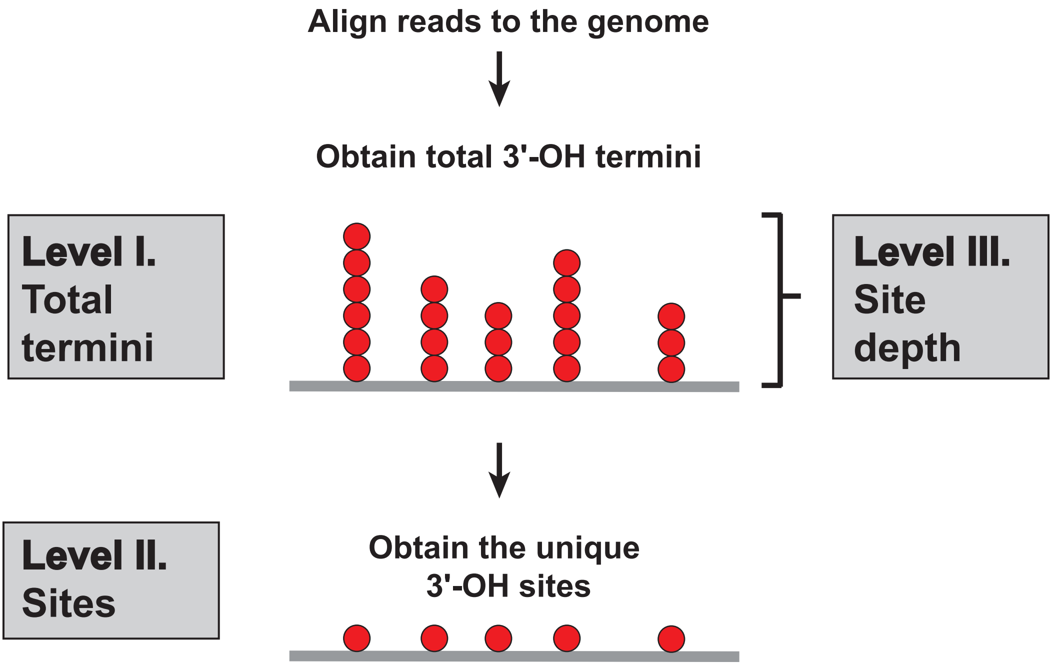

Figure S3. Analytical pipeline for calculation of total 3'-OH termini, 3'-OH sites with unique genomic coordinates and the depth of sites in the mitochondrial genome.

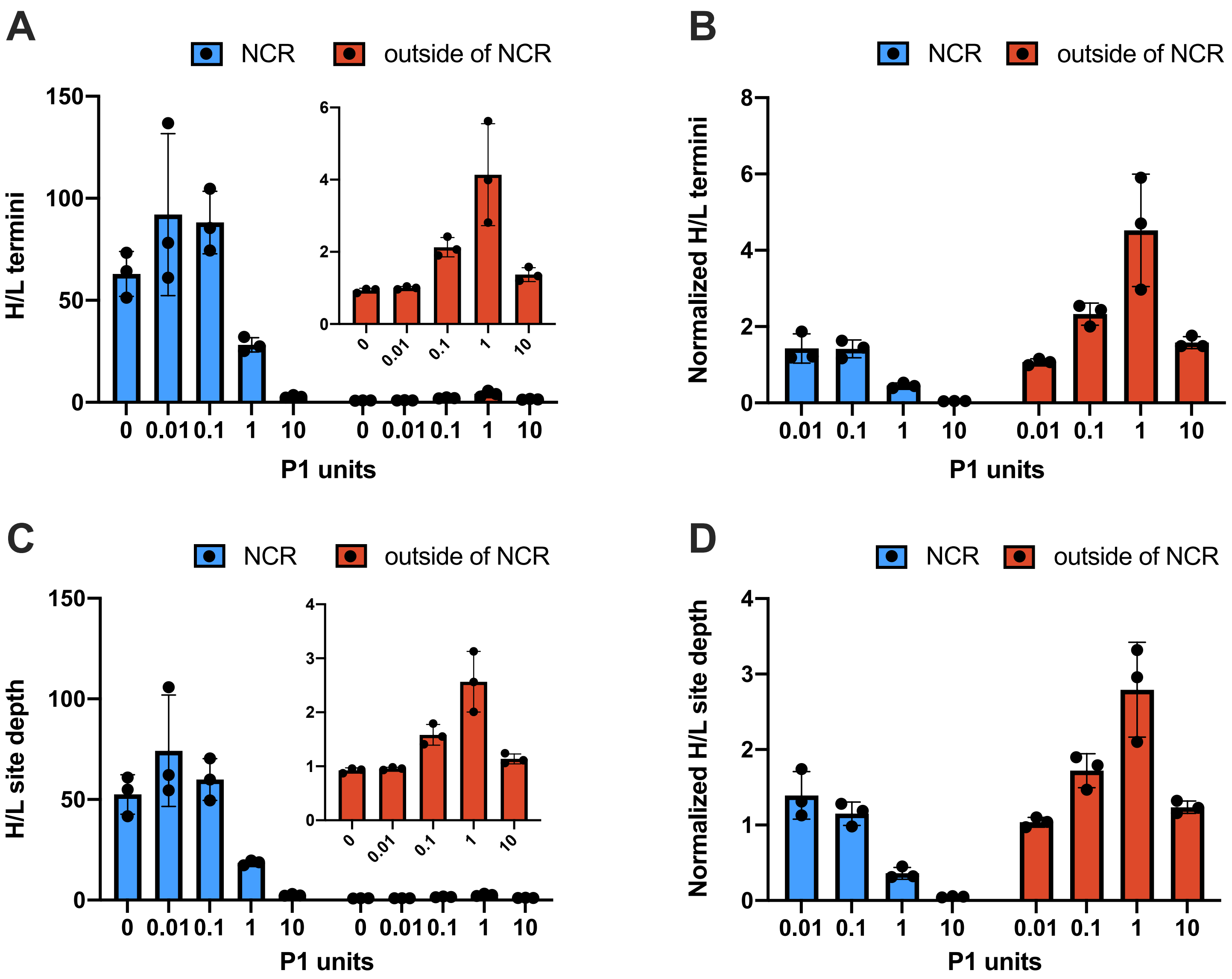

Figure S4. Analysis of the H vs. L strand distribution of essDNA signal using additional metrics. (A, B) The H/L ratios (A) and H/L normalized ratios (B) calculated using total 3'-OH termini. (C, D) The H/L ratio (C) and H/L normalized ratio (D) using the depth of sites. The normalized ratios were obtained by dividing the H/L ratios of the P1 treated samples to the H/L ratio of the untreated control. The error bars indicate SD of three independent biological replicates.
